# New Method to Calculate the Angular Weighting Function for a Scattering Instrument: Application to a Dust Sensor on Mars

**DOI:** 10.3390/s22239216

**Published:** 2022-11-27

**Authors:** David Santalices, Antonio J. de Castro, Susana Briz

**Affiliations:** 1 LIR—Infrared Laboratory, Department of Physics, Universidad Carlos III de Madrid, 28911 Leganés, Spain; 2 Science Faculty, Universidad Nacional de Educación a Distancia (UNED), 28040 Madrid, Spain

**Keywords:** scattering sensor, nephelometer, angular weighting function, scattering of particles

## Abstract

Suspended dust above the Martian surface is an important element in Martian climatology. In the frame of the Exomars’22 mission, we developed a dust sensor instrument, designed to provide size parameters of dust particles suspended in Mars surface from the light scattered by the particles. Thus, to interpret the data of the dust sensor, we need a method to calculate the theoretical optical power dispersed by the particles and, therefore, the theoretical signal obtained by the instrument. This signal depends on the suspended particles and on the instrument configuration. In this paper, we present a new method to calculate the angular weighting function ($W_{f}$) for scattering sensors. $W_{f}$ encompasses the scattering angles measured by the sensor and depends only on the instrument and not on the suspended particles. To calculate this $W_{f}$, we use fundamental radiometry principles and an appropriate coordinate system, where one coordinate is the scattering angle. The method is applied to the dust sensor instrument and compared with other methods. The comparison highlights the advantages of the proposed method since it avoids using an ideal sampling volume, preserves the radiometric meaning, and avoids instrument calibration. The effectiveness of the method makes it a valuable tool for the design of scattering instruments and also for the interpretation of their data.

## 1. Introduction

The characterization of suspended particles is necessary in different disciplines, such as environmental science [1], oceanography [2], planetary exploration [3], and so forth. This characterization can be performed by measuring the light scattering of such particles and, more specifically, their inherent optical properties (IOPs).

This is the case of the dust sensor (DS) [4], a mini-instrument whose scientific objective is the measurement of the size parameters of suspended dust and has been integrated into the meteorological suite METEO at the Surface Platform of the ExoMars’22 mission (currently canceled).

Suspended particles play a key role in the meteorology and climate of Mars. Thus, determining the effective dust parameters is important for radiometric modeling of the planet [5]. Over the past 50 years, many instruments (both orbiters and in-situ instruments [6]) have measured the opacity of the Martian atmosphere in different spectral ranges and conditions, resulting in multiple studies of dust size parameters [7,8,9,10].

Currently, there is an increasing interest in in situ instruments to study the surface–atmosphere interactions [11,12]. ESA is also considering a comprehensive network of weather landers on its flagship mission to Mars in the 2030s [13]. One of the desirable requirements for the instruments of such networks would be low cost–weight–size, that is, their miniaturization.

The DS instrument is designed to provide local measurements of near-surface dust scattering, meeting those miniaturization requirements.

**Figure 1:**
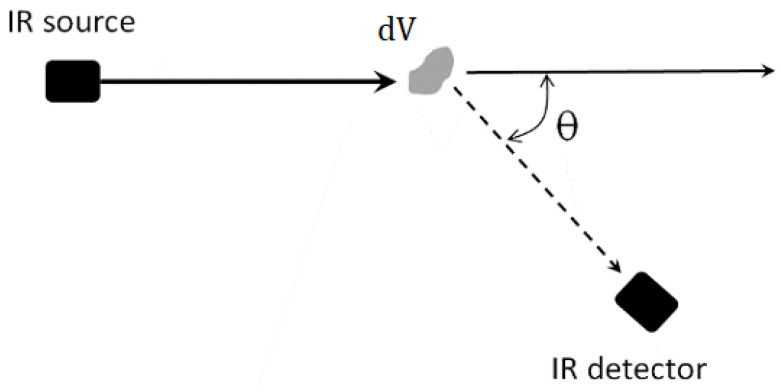
A scheme showing the scattering process.

The physical principle of measurement is based on the fact that light scattered by a particle depends on its size, shape, the incident light wavelength, and the angle that light is scattered, θ (Figure 1). Therefore, by measuring the scattered light in different directions and wavelengths, it is possible to retrieve information about the dust size parameters.

**Figure 2:**
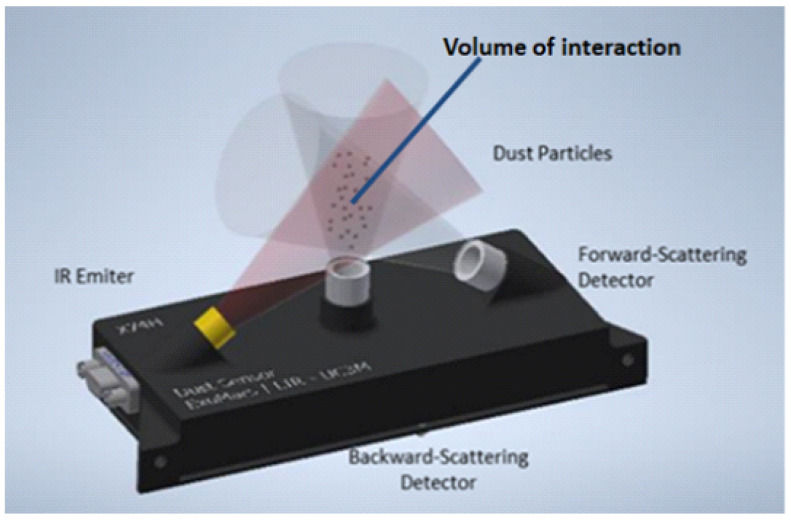
Dust sensor scheme.

According to the physical principle, our DS instrument consists of an IR source that emits infrared light and two detectors that measure the light scattered by the particle cloud in two spatial directions (Figure 2): forward-like and backward-like.

One can describe the light scattered by a cloud of particles (in terms of intensity) by considering the individual scattering of each particle in a volume. However, some instruments can relate their signal to the volume scattering function (β [$m^{-1}{\mathrm{sr}}^{-1}$]), an IOP describing the angular distribution of the scattered light by an infinitesimal volume element when an unpolarized plane wave illuminates it (Figure 1).

(1) dI=E·β·dV

where *E* [${W/m}^{2}$] is the incident irradiance, and (dI [W/sr]) is the radiant intensity scattered by a volume element (dV [$m^{3}$]).

Thus, to interpret the DS measurements, it is essential to relate the signal received by the detectors to the IOPs of the media, in this case β. Since these instruments span a wide range of scattering angles, we can express the optical flux received by the detectors, $\Phi_{D}$, as an integral of the β multiplied by an angular weight function ($W_{f}$) over all scattering angles:

(2) $$\Phi_{D}=\int_{0^{\circ }}^{180^{\circ }}\beta \left(\lambda ,\theta \right)\cdot W_{f}\left(\theta \right)d\theta$$

$W_{f}$ describes an instrument’s angular response (in terms of scattering angles) and it depends on the geometry and optics of the instrument. This $W_{f}$ in a polar nephelometer, for example, can be approximated to a Dirac delta ($W_{f}=\delta \left(\theta \right)$) [14]. Another example would be an integrating nephelometer, which presents a sinusoidal response ($W_{f}=\sin\theta$). Some studies include a corrected $W_{f}$ (called angular sensitivity function) to solve the non-idealities [15,16,17].

Regarding backward/forward instruments, their $W_{f}$ depends mostly on the sampling volume. The sampling volume is the region defined by the particles that scatter light that is measured by the detector and is related to $W_{f}$ because it indicates the range of scattering angles that the instrument ‘sees’. This volume is commonly defined by the intersection between two cones, the beam divergence of the source, and the detector’s field of view (FoV). Thus, the sampling volume defines an ideal region where any scattering contribution outside of it is not considered. Although this assumption is valid for collimated sources and detectors, it is less good when such elements are not highly collimated. In backscatter sensors, some studies compute $W_{f}$ by partitioning the ideal sampling volume into elementary volume elements [18,19]. In any case, the definition of the sampling volume is the main handicap and the main source of uncertainty of all these methods to calculate $W_{f}$, either because they consider just an ideal volume or a correction to resolve non-idealities.

In this work, we present a new method to compute $W_{f}$ that overcomes the drawbacks associated with the volume definition. Indeed, this method avoids using the ideal sampling volume; instead, we defined the volume weighting function (*VWF*), a function which encompasses the instrumental response in each spatial location, and that considers the instrument geometry, the source emission pattern, and the angular sensitivity (rather than the divergence and FoV).

The definition of a specific coordinate system, where one of its coordinates is the scattering angle, allows us to compute $W_{f}$ using the *VWF*. This $W_{f}$ preserves a radiometric meaning; thus, a proper characterization of the optical elements (source and detector) avoids the instrument calibration. In this way, a direct model of the instrument signal is defined, where the instrumental part ($W_{f}$) is separated from the scattering of the particles (β).

Furthermore, we used this method to calculate the $W_{f}$ of the DS instrument. The required geometric information and the characterization of the optical elements were obtained experimentally.

After calculating $W_{f}$ for the DS instrument, it was validated with the results of the Monte Carlo method, and with $W_{f}$ calculated by another method used for other commercial sensors [19].

In Section 2, we describe the method used to calculate $W_{f}$. In Section 3, we present $W_{f}$ obtained for the dust sensor. The validation, also in Section 3, includes a comparison of the optical power with a Monte Carlo Method and a comparison of the normalized $W_{f}$ obtained in [19]. In Section 4, we discuss some relevant aspects of this method.

## 2. Method and Data

**Figure 3:**
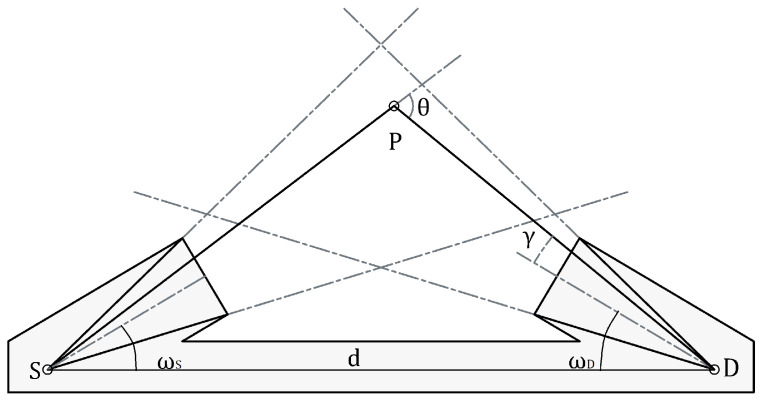
Schema showing the geometry of the problem. S: Source. D: Detector. P: Point where is located the scattering element. θ: Scattering angle. γ: Angle between optical axis and P ($\gamma_{S}$ for S and $\gamma_{D}$ for D). ω: angle between $\bar{SD}$ and the optical axis.

This method relies on the fact that for a fixed position of a detector (D) and a source (S), each point (P) is associated with a single scattering angle (Figure 3).

An appropriate choice of the coordinate system reduces the problem from a triple integral to a simple integral. In addition, this approach allows us to find the power received by each detector and quantify the distinct scattered angles detected by the instrument.

The reasoning followed in the next subsections should take into account the light spectral dependence. However, for the sake of clarity, this dependence was omitted.

### 2.1. Radiant Flux of the Detector

Using the definition of β (Equation (1)), the radiant flux ($\Phi_{D}$ [*W*]) reaching the detector can be calculated as

(3) $$d\Phi_{D}=E\cdot \beta \cdot dV\cdot \frac{A\cdot \sigma }{r^{2}}$$

where *A* is the area of the detector, *r* is the distance between dV and the detector, and σ is the angular sensitivity of the detector.

The term $\left(A\cdot \sigma \right)/r^{2}$ [sr] is the solid angle underlying between dV and the detector and, in addition, takes into account the angular response of the detector. Thus, for an ideal detector, σ equals to cos(γ) and the term turns into the projected solid angle ($A\cdot \cos\left(\gamma \right)/r^{2}$).

Equation (3) shows that the signal received by the detector depends on the β and the instrument’s characteristics (source irradiance, etc.). Thus, we can define a new function that encompasses all the parameters that appear in Equation (3), which depend only on the instrument and not on the particles. This function, which we call volume weighting function (*VWF*), is defined as follows:

(4) $$VWF=E\cdot \frac{A\cdot \sigma }{r^{2}}$$

Therefore, the total flux reaching the detector can be written as follows:

(5) $$\Phi_{D}={\int \int \int }_{V}dx\;dy\;dz\;VWF\left(x,y,z\right)\cdot \beta \left(\theta \left(x,y,z\right)\right)$$

In the next section, we will see how an appropriate coordinate system selection allows us to find $W_{f}$ using the *VWF*.

### 2.2. Angular Weight Function

Comparing Equation (2) and (5), one can see that, by choosing an appropriate coordinate system in which one of the coordinates is θ, β can be expressed as an exclusive function of θ.

(6) $$\Phi_{D}={\int \int \int }_{V}d\theta \;d\phi \;d\alpha \;J\left(\theta ,\alpha \right)VWF\left(\theta ,\phi ,\alpha \right)\cdot \beta \left(\theta \right)$$

where J(θϕα) is the Jacobian determinant. The new coordinate system (θ,ϕ,α) is defined in Appendix A.

This is why a suitable coordinate system allows one to find $W_{f}$ from the *VWF*. Since *VWF* would be a function of θ and two coordinates more, $W_{f}$(θ) can be understood as the surface integral of the *VWF* over a scattering angle isosurface:

(7) $$W_{f}\left(\theta \right)={\int \int }_{\Theta }dS\;VWF$$

where Θ is the surface that contains all the points with the same scattering angle.

Thus, the new coordinate system allows us to parameterize the surface, Θ, and therefore calculate $W_{f}$ from the *VWF*. Thus, $W_{f}$ in this coordinate system is defined as

(8) $$W_{f}\left(\theta \right)=\int_{-90^{\circ }}^{+90^{\circ }}d\phi \int_{-90^{\circ }}^{+90^{\circ }}d\alpha \;J(\theta ,\alpha )\mathrm{VWF}(\theta ,\phi ,\alpha )$$

Moreover, the proposed method to calculate $W_{f}$ also allows us to calculate an angular weighting function using the sampling volume ($W_{f,\;ideal}$) instead of the *VWF*, simply by inserting a mask delimiting the volume. Precisely, to compare both angular weighting functions, a mask is introduced in Equation (8):

(9) $$W_{f,\;ideal}\left(\theta \right)=\int_{-90^{\circ }}^{+90^{\circ }}d\phi \int_{-90^{\circ }}^{+90^{\circ }}d\alpha \;J(\theta ,\alpha )\cdot M(\theta ,\phi ,\alpha )\cdot \frac{A\cdot \cos(\gamma (\theta ,\phi ,\alpha ))}{r^{2}(\theta ,\phi ,\alpha )\cdot r_{\mathrm{SP}}^{2}(\theta ,\phi ,\alpha )}$$

where $r_{SP}$ is the distance between S and P, and M is a logical function equal to one where the point is inside the sampling volume and equal to zero outside it:

(10) $$M\left(\theta ,\phi ,\alpha \right)=\left\{\begin{array}{cc} 1 & \mathrm{if}\;(\theta ,\phi ,\alpha )\in \;\mathrm{sampling}\;\mathrm{volume}\;\\ 0 & \mathrm{if}\;(\theta ,\phi ,\alpha )\notin \;\mathrm{sampling}\;\mathrm{volume}\; \end{array}\right.$$

In Equation (9), the term $1/r_{SP}^{2}$ takes into account the quadratic decay of the source. However, it is a term that should not be considered with sources that do not show a quadratic decay (such as a laser). The term $A\cdot \cos(\gamma )/r^{2}$ is the projected solid angle between the dV and the detector.

### 2.3. Data

**Figure 4:**
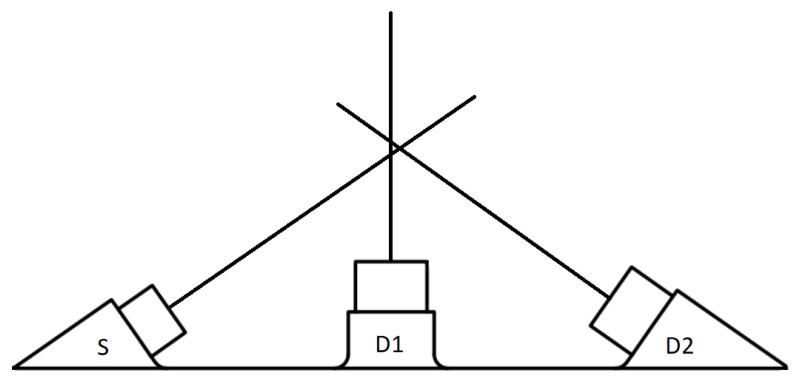
Schema of the dust sensor. S refers to the source. D1 and D2 refer to the backward and forward detectors, respectively.

We applied the method to calculate $W_{f}$ explained in the previous subsection to the DS instrument [4], consisting of an IR source (S) and a forward (D1) and backward (D2) detectors located at specified scattering angles (Figure 4). The geometric parameters (positions and orientations of the emitter and the detector) for both configurations can be found in Appendix B. Each detector encompasses two different spectral bands in the range of 1–3 μm and 3–5 μm. The thermal source emits in a similar way to a blackbody with a peak emission of 3.29 μm. To avoid thermal noise, the instrument implements lock-in amplification [20].

$W_{f}$ and $W_{f,\;ideal}$ use different inputs for their calculation. That is, $W_{f}$ requires knowing the irradiance pattern of the emitter, *E*, and the angular sensitivity of the detector, σ. However, to compute $W_{f,\;ideal}$, we need to know the sampling volume given by the detector FoV and the emitter divergence.

The irradiance pattern (E(x,y,z)) and the angular sensitivity of the detector (σ(x,y,z)) were fitted to the parametric functions using empirical data. Equations (A2) and (A3) correspond to the E(x,y,z) and σ(x,y,z) used to calculate $W_{f}$.

The detector FoV and emitter divergence are calculated by applying a full width at half-maximum criterion to E(x,y,z) and σ(x,y,z). Both of them are used to calculate the sampling volume mask, M, used to calculate $W_{f,\;ideal}$.

To compute both angular weighting functions, we define a mesh of 200 × 200 × 200 nodes for the coordinates (θ, ϕ, α). Then, we perform the numerical integration of the Equations (8) and (9). The software developed to calculate $W_{f}$ and $W_{f,\;ideal}$ for any geometry, E(x,y,z) and σ(x,y,z) can be found in the data section of this paper.

As this work is about determining a method, and its theoretical application, all the radiometric constants involved, such as the power or the sensitivity of the source or detector, were normalized.

## 3. Results

### 3.1. Angular Weight Functions of the Dust Sensor

**Figure 5:**
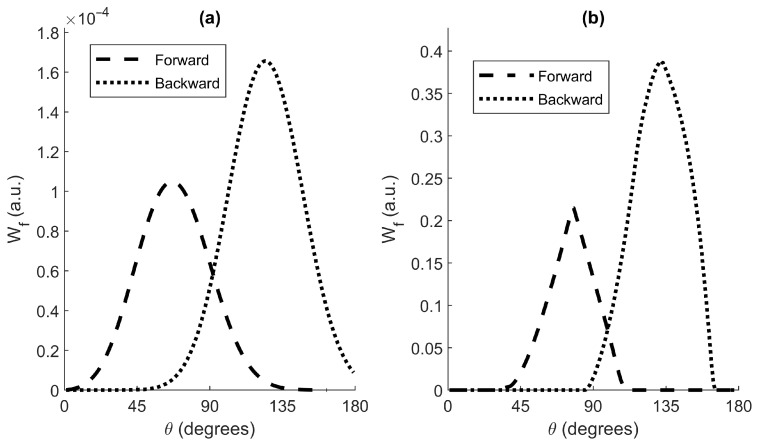
Angular weighting functions of the dust sensor for both configurations as a function of the scattering angle (θ). (**a**) $W_{f}$ using Equation (8). (**b**) $W_{f,ideal}$ using the Equation (9).

Figure 5a represents $W_{f}$ of the dust sensor instrument for both configurations. The backward configuration shows a peak at 125${}^{\circ }$ (coincident with its nominal scattering angle, $\theta_{N}$, see Appendix B). The forward configuration shows a peak at 66${}^{\circ }$ (a lower value than its $\theta_{N}$). Both configurations present a wide angular range (from 90${}^{\circ }$ to 160${}^{\circ }$ and from 30${}^{\circ }$ to 110${}^{\circ }$).

Figure 5b represents $W_{f,ideal}$. Compared to Figure 5a, we can see how the angle span observed by the instrument when considering an ideal volume, instead of the *VWF*, is smaller.

One may notice the relative size between the angular weighting functions of the forward and backward configurations. Although the source and the detectors are the same for both configurations, the quadratic decay behavior of light intensity is reflected in the longer source-detector path existing for the forward configuration (Figure 4).

### 3.2. Validation

#### 3.2.1. Monte Carlo

This method was compared against the Monte Carlo method. For that purpose, we performed the following simulation:

We create a random distribution of spherical particles. Then, using the detector-source configurations of Section 3.1, we calculate the spectral radiant flux on each particle and the radiant flux that each particle contributes to the detector:

(11) $$\Phi_{D}=\sum_{j=1}^{N}E_{i}\left(x_{j},y_{j},z_{j}\right)\frac{S_{11}\left(\theta_{j},R,m\right)}{k^{2}}\frac{A\cdot \sigma (x_{j},y_{j},z_{j})}{r^{2}\left(x_{j},y_{j},z_{j}\right)}$$

where $S_{11}$ is the first element of the scattering matrix, *k* is the wavenumber, *m* is the complex refractive index, and *R* is the particle radio.

For the Monte Carlo simulation, we evaluate the Equation (11) for the forward/backward configurations. For this purpose, we create a random distribution of 80,000 spherical particles in a volume of 0.016 m${}^{3}$, giving a density of 5 particles/cm${}^{3}$ (conditions expected in the Martian atmosphere [21]). Then, the resulting radiant flux from each detector is averaged over 200 simulations. The simulations are performed using a 1 μm wavelength and for particles with a radius of 0.25 to 5 μm. The refractive index used is: m=1.5+0i.

In this paper, Mie’s theory is used to validate the method. However, depending on the wavelength, this assumption may be an important source of error [22,23]. For experimental comparisons or to solve the inverse problem, the non-sphericity of the particles should be addressed using, for example, the T-matrix method [24].

To calculate the radiant flux using $W_{f}$, we evaluate the Equation (2) using the β of the concrete particle distribution.

**Figure 6:**
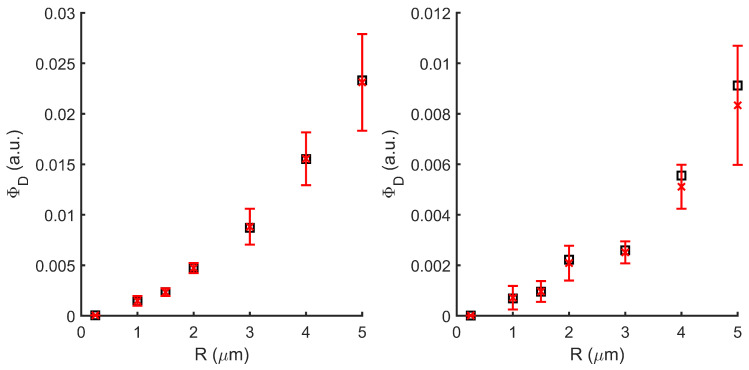
Detector intensity comparison between the Monte Carlo (red bar error) and $W_{f}$ (black squares) methods for different particle radius. **Left**: forward, **right**: backward.

The comparison (Figure 6) between the Monte Carlo (red errorbars) and $W_{f}$ (black squares) shows great agreement between both methods.

#### 3.2.2. Commercial Backscattering Sensors

Zhang et al. [19] applied a method to compute the normalized $W_{f,ideal}$ for two commercial backscatter sensors: the HydroScat and the Eco-BB. In order to compare both methods, we calculated the $W_{f,ideal}$ using the algorithm described in this paper for both sensors.

**Figure 7:**
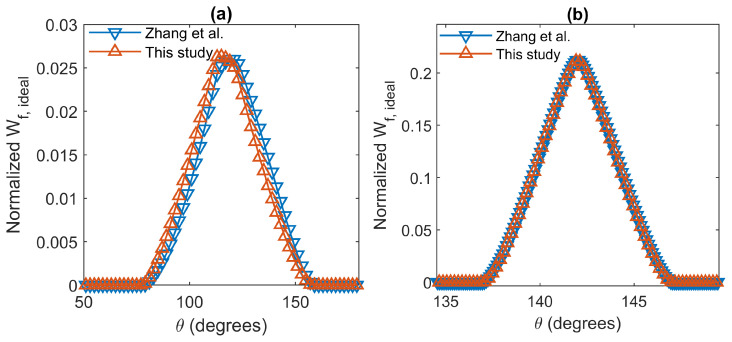
Comparison between the normalized $W_{f,ideal}$ using different methods, θ is the scattering angle [19]. (**a**): Eco-BB. (**b**) HydroScat.

Figure 7 show this result. The $W_{f,ideal}$ calculated by both methods are practically identical in the hydroScat instrument, with a similar peak value and shape. In the case of the Eco-BB, which encompasses a wider range of angles, the shape is similar, but the peak value is slightly different in both methods. These results are coherent since the method presented by Zhang et al. [19] applies an approach that is more precise for sensors that encompass a narrow range of scattering angles.

## 4. Discussion

In this section, we discuss the results, the validation, and some relevant aspects related to this method, such as light attenuation, multiple scattering, geometrical constraints, or instrument calibration.

### 4.1. Results and Validation

The analysis of $W_{f}$ reveals that their peaks are very similar to the nominal scattering angles. Nevertheless, the forward $W_{f}$ peak is smaller than the nominal scattering angle. This leads us to think that the $W_{f}$ calculation can also be useful for detecting behaviors of the instrument elements that are diverted from the nominal. The comparison of $W_{f}$ and $W_{f,ideal}$ makes it clear that the last one neglects scattering angles, θ, that really contribute to the detector’s signal.

The comparison of the signal obtained with the Monte Carlo method shows that $W_{f}$ maintains a radiometric sense, justifying the use of volume elements rather than particles. The advantage of this method over Monte Carlo is its ability to group the instrumental response into one term, $W_{f}$. The advantage of using $W_{f}$ is twofold. First, it allows us to develop inversion algorithms to estimate b, and second, it can be used to calibrate the system. In addition, being able to separate the geometrical part is very valuable in the design and optimization of the instrument.

In addition to the validation by the Monte Carlo method, we compared the angular function with that of another method when a sampling volume is taken into account ($W_{f,ideal}$). Furthermore, we saw that the use of a sampling volume results in a truncated angular weighting function. In this method, since it is not necessary to use a sampling volume, we obtain a $W_{f}$ that includes all the scattering angles.

### 4.2. Light Attenuation

The algorithm proposed in this work to calculate $W_{f}$ does not take into account the attenuation suffered by the light along its path. This is because the DS is intended to operate on Mars, where the expected particle density is low. However, this factor may become important when the medium where the particles are located significantly attenuates the light, either because of high particle densities or because of the presence of other elements that may attenuate the light (gases, water, etc.).

This factor, in principle, can be taken into account during the $W_{f}$ calculation. Taking into account the Beer–Lambert law, which describes the attenuation of light traveling through a medium, one can write Equation (3) as

(12) $$d\Phi_{D}=E\cdot e^{c\cdot r_{SD}}\beta \cdot dV\frac{A\sigma }{r^{2}}e^{c\cdot r}=VWF\cdot \beta \cdot dV\cdot e^{c(r_{SD}+r)}$$

where *c* is the absorption coefficient of the medium. The term $r_{SD}+r$ indicates the total light path from the emitter to the detector.

In this way, $W_{f}$ can be calculated taking into account the attenuation of the light:

(13) $$W_{f}\left(\theta \right)=\int_{-90^{\circ }}^{+90^{\circ }}d\phi \int_{-90^{\circ }}^{+90^{\circ }}d\alpha \;J(\theta ,\alpha )\mathrm{VWF}(\theta ,\phi ,\alpha )e^{c(r_{\mathrm{SD}}+r)}$$

The fact that $W_{f}$ depends on the absorption coefficient of the medium is definitely a disadvantage since the idea of $W_{f}$ is to separate the instrumental response from the β. When the attenuation of the medium can be neglected, $W_{f}$ only depends on the instrument. However, when the attenuation of the medium cannot be neglected, $W_{f}$ becomes dependent on the attenuation coefficient, which makes it a function that depends not only on the instrument, but also on the medium.

**Figure 8:**
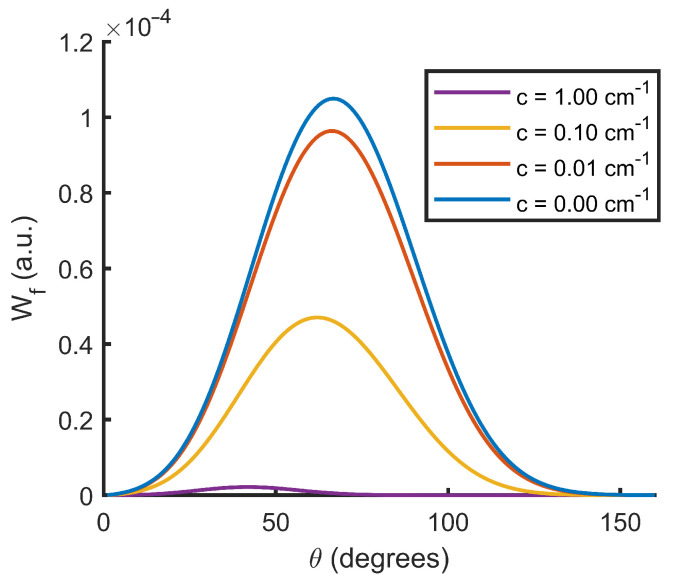
Angular weighting function ($W_{f}$) as a function of the scattering angle (θ) for different attenuation coefficients (*c*).

Figure 8 is an example of this effect. In it, we can observe how an increase in the attenuation coefficient decreases the value of $W_{f}$. In addition, we can observe how the peak value of the function shifts toward smaller scattering angles because, in general, the points associated with a smaller scattering angle have a shorter optical path.

### 4.3. Spectral Dependence

In the calculation of W in this work, its spectral dependence is not taken into account. When the emitter is not monochromatic, it is necessary to consider this dependence. The optical flux reaching the detector would be given by

(14) $$\Phi_{D}=\int_{0}^{\infty }d\lambda \int_{0}^{180^{\circ }}d\theta \;W_{f}\left(\theta ,\lambda \right)\beta \left(\theta ,\lambda \right)$$

It should be noted that this W implies the emission pattern of the source, as well as the spectral dependence of the source. In principle, this will cause changes only in the magnitude of $W_{f}$, but not in its shape. In such cases,

(15) $$W_{f}\left(\lambda ,\theta \right)=f\left(\lambda \right)\cdot g\left(\theta \right)$$

We can express $W_{f}$ as the product of a spectral function, *g* and an angular function, *f*; in other words, the instrument “sees” the same scattering angles for all wavelengths but with different magnitudes.

There are other situations where this is not true, for example, the existence of a medium where the absorption coefficient has a spectral dependence or interferential filters, where the spectral sensitivity depends on the viewing angle.

### 4.4. Single Scattering

The method for finding $W_{f}$ presented in this document was computed using a first-order scattering approximation [25]. This assumption implies that the particles only scatter the light coming from the emitter (ignoring the scattered light from other particles). Thus, the lower the particle density of the medium, the more reliable the $W_{f}$ computed by this method [26]. A multiple-scattering regime can produce secondary peaks in the $W_{f}$ or produce a change in the shape of the primary peak, as shown in [19].

### 4.5. Geometric Constraints

As we saw in Section 3.2, an $W_{f}$ can be calculated with the concept of sampling volume. To do so, we added a binary mask in Equation (9), indicating the space where a scattering event can or cannot be measured. However, this is just an example of an advantage of our method to calculate $W_{f}$. In general, many changes in the geometrical configuration can be modeled with a geometrical mask.

For instance, it is possible to add a geometric constriction mask to the *VWF* that limits the areas where scattering may or may not be measured. Specifically, the emitters and detectors of such instruments are usually contained within an encapsulation (which makes it impossible to find particles in those locations). Thus, it is possible to obtain truncated $W_{f}$s by adding a mask, $M_{Tr}$, to Equation (8):

(16) $$W_{f}\left(\theta \right)=\int_{-90^{\circ }}^{+90^{\circ }}d\phi \int_{-90^{\circ }}^{+90^{\circ }}d\alpha \;J(\theta ,\alpha )\mathrm{VWF}(\theta ,\phi ,\alpha )M(\theta ,\phi ,\alpha )$$

**Figure 9:**
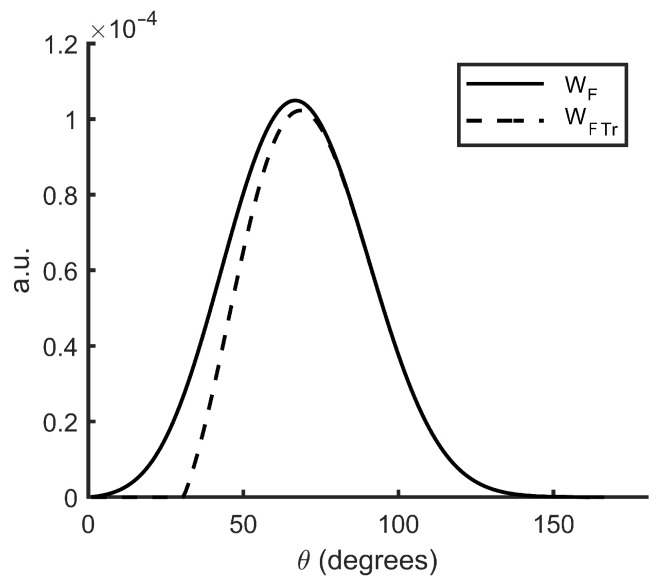
Comparison between the angular weighting function ($W_{f}$) and the truncated angular weighting function as a function of the scattering angle (θ).

Figure 9 shows a truncated $W_{f}$ of the forward DS configuration. For this purpose, a geometric mask was placed for all positions z < 10 mm. We can see how a geometric constraint to the particles translates to a reduction of the scattering angles that the instrument can ‘see’.

### 4.6. Calibration

Although this method theoretically does not require calibration (as long as the emitter and detector are well-characterized radiometrically), in practice, an experimental calibration encompasses the optical and electronic response in a constant factor. According to the literature, there are two ways to calibrate this kind of sensor [18].

One method relies on the use of different media with a well-characterized phase function (or β). This approach makes sense for marine sensors, as a tank can be filled with a liquid with a given β. However, this method can be a problem for atmospheric sensors, where it is more difficult to create a controlled environment.

**Figure A1:**
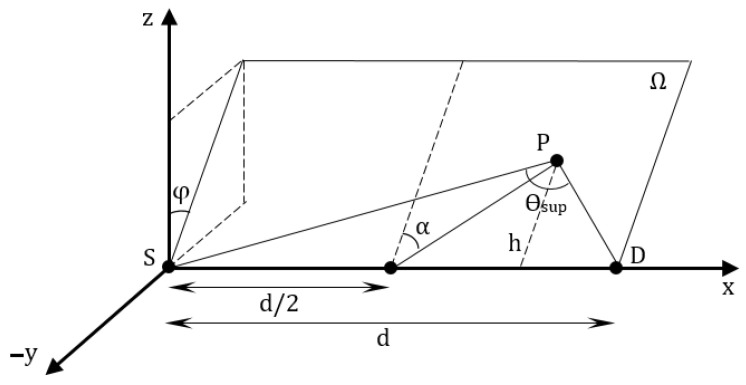
Coordinate system used to express each point P as a function of the angles α, ϕ and $\theta_{sup}$.

The other method calibrates the response of the sensor using a Lambertian target with a known reflectivity. Maffione and Dana [27] present a mathematical derivation of this method using Lambertian planes at different profundities and relate the profundity with $W_{f}$. This approach is valid for backscattering sensors, where the sampling volume comprises a narrow range of scattering angles. Instruments with large conical sections comprise a range of scattering angles. The relationship between the scattering angle and the Cartesian coordinates (Equation (A1)) is given by the inscribed angle theorem. Giving a fixed position of S and D (Figure A1), all the scattering angles with the same value are contained in a convoluted arc circle. Thus, to correctly relate the profundity weight function to the $W_{f}$, it would be necessary to use convoluted arc circles surfaces rather than flat plaques.

**Figure 10:**
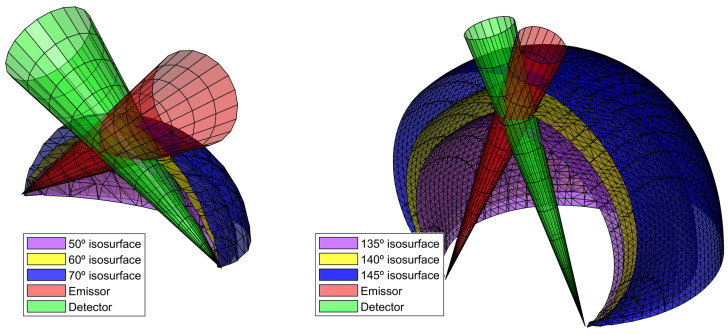
Scattering angleisosurfaces for different configurations.

Figure 10 shows the different scattering angle isosurfaces (for a fixed S and D) and the ideal (conical) FoV of the emitter and the detector. The intersection volume between the detector and emitter cones shows the volume where the β is measured. The smaller the volume of the intersection, the more similar the isosurfaces will be to the planes (within that volume).

## 5. Conclusions

In this work, we developed a method to compute a $W_{f}$ that allows us to obtain the radiant flux that an instrument would receive from the light scattered by a distribution of suspended dust particles. In addition, we applied the method to a dust sensor designed to measure the properties of the dust particles suspended in the Martian atmosphere. Finally, we compared our method with others based on different principles (Monte-Carlo-based) and applied it to other commercial sensors.

The main advantage of our proposal is that the method does not require the previous definition of a sampling volume. On the contrary, our method considers the contribution of all particles, thus avoiding uncertainties due to idealizations and approximations made when defining the sampling volume.

Another strength of our method lies in the fact that, thanks to an appropriate choice of coordinates, it is able to isolate the contribution of the geometric parameters of the instrument from the optical properties of the particles. This feature is of vital importance when designing scattering instruments since it allows to simulate the signal received by the detector in a much more agile, efficient and fast way. Thus, the method proposed in this work reduces the computational and time cost of simulating different geometric designs of the instrument, especially when compared to methods based on particle-by-particle calculations.

The efficiency of the method is especially relevant to obtain physical information from the signal provided by the DS instrument. Indeed, the simulations carried out for different dust particle size distributions will provide relationships between the characteristics of the dust distributions and the signals received in the DS instrument. The derived algorithms will lead us to achieve the ultimate goal of the DS instrument: to determine the properties of the dust near the Martian surface from the signal of two detectors located in the forward and backward directions.

However, the scope of the $W_{f}$ calculation methodology goes beyond the DS instrument, as it can be applied to other scattering instruments and dust sensors in the Earth’s atmosphere and contribute to the knowledge of the meteorology and climatology of the planet.

## Figures and Tables

**Figure A2:**
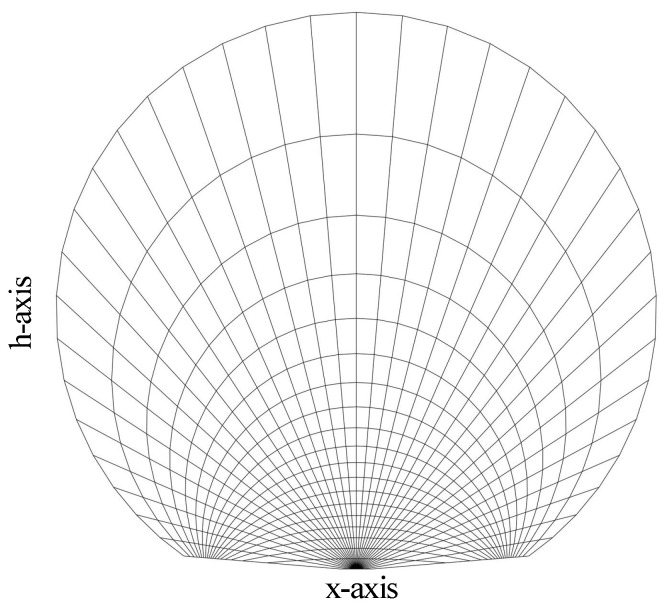
Coordinates grid for a fixed ϕ value.

## Data Availability

The software used to compute $W_{f}$ can be found at the following link: https://github.com/DavidSantalices/Angular-Weighting-Function (accessed on 20 November 2022).

## Appendix A. Coordinate System

In order to reduce the dimensions of the problem, it is needed to use a coordinate system where one of the coordinates is the scattering angle (θ).

Figure A1 shows a scheme of the new coordinate systems with the following:

- *x*, *y*, *z* are the Cartesian coordinates.
- *h* is the distance between P and the *x*-axis.
- *r* is the distance between P and D.
- S and D represents the source and detector points, respectively. Both contained in the *x*-axis.
- Ω is a plane that includes the *x*-axis; rotated with a ϕ angle from the *z*-axis. It also contains the P point and the r vector.
- *d* is the distance between S and D.
- $\theta_{sup}$ is the supplementary angle of the scattering angle ($\theta_{sup}=180-\theta$).
- α represents the complementary angle between the *x*-axis and the vector with it origen point at midpoint of the $\bar{\mathrm{SD}}$ line segment, and its terminal point at P.

Therefore, the following expressions show the relationship between both coordinate systems:

(A1) $$\begin{array}{cc} x & =\frac{d}{2}\pm d\tan\left(\alpha \right)\sqrt{\tan^{2}\left(\alpha \right)-\cos^{2}\left(\theta_{sup}\right)+1}\pm \frac{d\tan^{2}\left(\alpha \right)\cos\left(\theta_{sup}\right)}{2\tan\left(\alpha \right)\sin\left(\theta_{sup}\right)\left(\tan^{2}\left(\alpha \right)+1\right)}\\ y & =h\cdot \sin(\phi )\\ z & =h\cdot \cos(\phi ) \end{array}$$

where

$$h=\pm d\tan\left(\alpha \right)\sqrt{\tan^{2}\left(\alpha \right)-\cos^{2}\left(\theta_{sup}\right)+1}\pm \frac{d\tan^{2}\left(\alpha \right)\cos\left(\theta_{sup}\right)}{2\sin\left(\theta_{sup}\right)\left(\tan^{2}\left(\alpha \right)+1\right)}$$

The ± symbol indicates the piecewise character of the coordinates. Plus symbol for $\alpha \geq 0^{\circ }$. Minus Symbol for $\alpha <0^{\circ }$.

This coordinates are the result of the inscribed angle theorem, and indicate that θ isosurfaces have the shape of convoluted arc-circles. Figure A2 shows an example of the grid that creates the coordinates $\theta_{sup}$ and α for a fixed ϕ value. In this figure, we can appreciate the arc-circle $\theta_{sup}$ isolines.

This method relies on the definition of a coordinate system where one of its coordinates is the scattering angle (or related to it). However, the election of the other coordinates is not unique. Due to the cylindrical symmetry character of the scattering angle isosurfaces (Appendix A), the election of the ϕ coordinates seems natural. However, the α coordinate is the most likely to be changed. In this study, the α coordinate is an angle whose vertex coincides with the midpoint of $\bar{\mathrm{SD}}$ (Figure A1). This election creates a uniform grid around the plane normal to the segment $\bar{\mathrm{SD}}$, containing the $\bar{\mathrm{SD}}$ midpoint. A uniform grid around that zone is useful for symmetric sensors ($\omega_{S}\approx \omega_{D}$), which is the reason why we chose the alpha coordinate in this study.

## Appendix B. Emission Pattern, Angular Sensitivity and Geometry of the Dust Sensor

Table A1 contains the geometrical parameters of the DS, the Eco-BB, and the Hydroscat instruments.

**Table A1:** Geometrical parameters of different instruments. * [19]. ** The divergence and FoV of the DS was obtained using the full width at half maximum criteria of Equations (A2) and (A3).

Configuration	D (mm)	$\omega_{S}$ (${}^{\circ }$)	$\omega_{D}$ (${}^{\circ }$)	$\theta_{N}$ (${}^{\circ }$)	Divergence (${}^{\circ }$) **	FoV (${}^{\circ }$) **
Forward	72.8	35	35	70	32	48
Backward	36.0	31	94	125	32	48
Eco-BB ${}^{*}$	17.0	46.9	71.6	118.4	32	48
HydroScat ${}^{*}$	223	101.3	40.7	142	32	48

The irradiance pattern of the DS emitter was modeled using the following equation:

(A2) $$E\left(\gamma_{S}\right)=\frac{cte}{r_{SP}^{2}}\cos^{n}\left(\gamma_{S}\right)$$

where *n* and cte were calculated using experimental data.

The angular sensitivity of the detectors was modeled using the following equation:

(A3) $$\sigma \left(\gamma_{D}\right)=\sum_{i=1}^{3}a_{i}e^{-b_{i}{\left(\gamma_{D}-\gamma_{o,i}\right)}^{2}}$$

where $\gamma_{o,i}$ and $a_{i}$ were calculated using experimental data.

The emitter and detectors parameters can be found in Table A2.

Equations (A2) and (A3) are expressed in polar coordinates. Thus, it is necessary to express these equations in terms of (θ,ϕ,α). For this, it is necessary to transform the polar coordinates to Cartesian coordinates, rotate and translate the source and detector (using the parameters of Table A1), and use Equation A1 to change from Cartesian coordinates to the new coordinate system.

**Table A2:** Emitter and detector parameters.

Emitter			
***cte* (a.u.)**		***n***	
1		17	
**Detector**			
***i***	***a_i_***	***b_i_***	**γ*_o,i_***
1	0.62	0.0020	8.37
2	0.20	0.0350	0.00
3	0.62	0.0020	−8.37

## References

[1] World Meteorological Organization (2014). Guide to Meteorological Instruments and Methods of Observation.

[2] Jonasz M., Fournier G.R. (2007). Measurements of light scattering by particles in water. Light Scattering by Particles in Water.

[3] Falkner P., Peacock A., Schulz R. (2007). Instrumentation for Planetary Exploration Missions. Treatise on Geophysics.

[4] Russu A., de Castro A.J., Cortes F., López-Ongil C., Portela M., Garcia E., Miranda J.A., Canabal M.F., Arruego I., Martinez-Oter J. A light compact and rugged IR sensor for space applications. Proceedings of the Infrared Sensors, Devices, and Applications IX.

[5] Forget F., Montabone L. Atmospheric dust on Mars: A review. Proceedings of the 47th International Conference on Environmental Systems.

[6] Martínez G., Newman C., Vicente-Retortillo D., Fischer E., Renno N., Richardson M., Fairén A., Genzer M., Guzewich S., Haberle R. (2017). The modern near-surface Martian climate: A review of in-situ meteorological data from Viking to Curiosity. Space Sci. Rev..

[7] Tomasko M., Doose L., Lemmon M., Smith P., Wegryn E. (1999). Properties of dust in the Martian atmosphere from the Imager on Mars Pathfinder. J. Geophys. Res. Planets.

[8] Pollack J.B., Ockert-Bell M.E., Shepard M.K. (1995). Viking Lander image analysis of Martian atmospheric dust. J. Geophys. Res. Planets.

[9] Wolff M.J., Smith M.D., Clancy R., Spanovich N., Whitney B., Lemmon M.T., Bandfield J., Banfield D., Ghosh A., Landis G. (2006). Constraints on dust aerosols from the Mars Exploration Rovers using MGS overflights and Mini-TES. J. Geophys. Res. Planets.

[10] Chen-Chen H., Pérez-Hoyos S., Sánchez-Lavega A. (2019). Dust particle size and optical depth on Mars retrieved by the MSL navigation cameras. Icarus.

[11] Banfield D., Stern J., Davila A., Johnson S.S., Brain D., Wordsworth R., Horgan B., Williams R.M., Niles P., Rucker M. (2020). Mars Science Goals, Objectives, Investigations, and Priorities: 2020 Version.

[12] Diniega S., Barba N., Giersch L., Jackson B., Soto A., Banfield D., Day M., Doran G., Dundas C.M., Mischna M. It’s Time for Focused In Situ Studies of Planetary Surface-Atmosphere Interactions. Proceedings of the 2022 IEEE Aerospace Conference (AERO).

[13] ESA (2022). Terrae Novae 2030+ Strategy Roadmap.

[14] Malm W. (2016). Visibility: The Seeing of Near and Distant Landscape Features.

[15] Müller T., Nowak A., Wiedensohler A., Sheridan P., Laborde M., Covert D.S., Marinoni A., Imre K., Henzing B., Roger J.C. (2009). Angular Illumination and Truncation of Three Different Integrating Nephelometers: Implications for Empirical, Size-Based Corrections. Aerosol Sci. Technol..

[16] Moosmüller H., Arnott W.P. (2003). Angular truncation errors in integrating nephelometry. Rev. Sci. Instrum..

[17] Heintzenberg J., Wiedensohler A., Tuch T., Covert D., Sheridan P., Ogren J., Gras J., Nessler R., Kleefeld C., Kalivitis N. (2006). Intercomparisons and aerosol calibrations of 12 commercial integrating nephelometers of three manufacturers. J. Atmos. Ocean. Technol..

[18] Sullivan J.M., Twardowski M.S., Ronald J., Zaneveld V., Moore C.C. (2013). Measuring optical backscattering in water. Light Scattering Reviews 7.

[19] Zhang X., Leymarie E., Boss E., Hu L. (2021). Deriving the angular response function for backscattering sensors. Appl. Opt..

[20] Ramírez Bárcenas A., Paz Herrera R., Miranda Calero J., Canabal Benito M., García Ares E., Russu A., Cortés F., De Castro A., López F., Portela-García M. (2022). Optimized Design and Implementation of Digital Lock-In for Planetary Exploration Sensors. IEEE Sens. J..

[21] Kemmerer B.W., Lane J.E., Wang J.J., Phillips J.R., Johansen M.R., Buhler C.R., Calle C.I. (2021). Electrostatic precipitator dust density measurements in a Mars-like atmosphere. Part. Sci. Technol..

[22] Dabrowska D.D., Muñoz O., Moreno F., Ramos J.L., Martínez-Frías J., Wurm G. (2015). Scattering matrices of martian dust analogs at 488 nm and 647 nm. Icarus.

[23] Laan E., Volten H., Stam D., Munoz O., Hovenier J., Roush T. (2009). Scattering matrices and expansion coefficients of martian analogue palagonite particles. Icarus.

[24] Mishchenko M.I. (2000). Calculation of the amplitude matrix for a nonspherical particle in a fixed orientation. Appl. Opt..

[25] Mishchenko M.I., Hovenier J.W., Mackowski D.W. (2004). Single scattering by a small volume element. JOSA A.

[26] Mishchenko M.I., Liu L., Videen G. (2007). Conditions of applicability of the single-scattering approximation. Opt. Express.

[27] Maffione R.A., Dana D.R. (1997). Instruments and methods for measuring the backward-scattering coefficient of ocean waters. Appl. Opt..

